# Protection of natural infection against reinfection with SARS-CoV-2 JN.1 variant

**DOI:** 10.1093/jtm/taae053

**Published:** 2024-04-09

**Authors:** Hiam Chemaitelly, Peter Coyle, Mohamed Ali Ben Kacem, Houssein H Ayoub, Patrick Tang, Mohammad R Hasan, Hadi M Yassine, Asmaa A Al Thani, Zaina Al-Kanaani, Einas Al-Kuwari, Andrew Jeremijenko, Anvar H Kaleeckal, Ali N Latif, Riyazuddin M Shaik, Hanan F Abdul-Rahim, Gheyath K Nasrallah, Mohamed Ghaith Al-Kuwari, Adeel A Butt, Hamad E Al-Romaihi, Mohamed H Al-Thani, Abdullatif Al-Khal, Roberto Bertollini, Laith J Abu-Raddad

**Affiliations:** Weill Cornell Medicine-Qatar, Cornell University, Doha, Qatar; Hamad Medical Corporation, Doha, Qatar; Hamad Medical Corporation, Doha, Qatar; Department of Mathematics and Statistics and Department of Biomedical Science and Department of Public Health, Qatar University, Doha, Qatar; Department of Pathology, Sidra Medicine, Doha, Qatar; Department of Pathology and Molecular Medicine, McMaster University, Hamilton, Canada; Department of Mathematics and Statistics and Department of Biomedical Science and Department of Public Health, Qatar University, Doha, Qatar; Department of Mathematics and Statistics and Department of Biomedical Science and Department of Public Health, Qatar University, Doha, Qatar; Hamad Medical Corporation, Doha, Qatar; Hamad Medical Corporation, Doha, Qatar; Hamad Medical Corporation, Doha, Qatar; Hamad Medical Corporation, Doha, Qatar; Hamad Medical Corporation, Doha, Qatar; Hamad Medical Corporation, Doha, Qatar; Department of Mathematics and Statistics and Department of Biomedical Science and Department of Public Health, Qatar University, Doha, Qatar; Department of Mathematics and Statistics and Department of Biomedical Science and Department of Public Health, Qatar University, Doha, Qatar; Primary Health Care Corporation, Doha, Qatar; Hamad Medical Corporation, Doha, Qatar; Ministry of Public Health, Doha, Qatar; Ministry of Public Health, Doha, Qatar; Hamad Medical Corporation, Doha, Qatar; Ministry of Public Health, Doha, Qatar; Weill Cornell Medicine-Qatar, Cornell University, Doha, Qatar

**Keywords:** COVID-19, reinfection, case-control, test-negative, immunity, epidemiology

## Abstract

Overall effectiveness of infection in preventing reinfection with SARS-CoV-2 JN.1 variant was estimated at 1.8% (95% CI: −9.3to 12.6%), and demonstrated rapid decline over time since the previous infection, decreasing from 82.4% (95% CI: 40.9 to 94.7%) within 3 to less than 6 months, to a negligible level after one year.

Evidence at the level of neutralizing antibodies suggests that the SARS-CoV-2 JN.1 variant demonstrates increased immune evasion compared to its parent lineage BA.2.86 and to recently circulating variants, such as XBB.1.5 and EG.5.1.^1^ JN.1 has also exhibited a growth advantage over other variants and triggered large SARS-CoV-2 waves in various countries,^2^ prompting the World Health Organization to classify it as a variant of interest on 19 December 2023.^2^ We estimated the effectiveness of natural infection in preventing reinfection with JN.1 during a large JN.1 wave in Qatar using the test-negative case-control study design.^3^^,^^4^

Qatar's national COVID-19 databases were analysed between 4 December 2023, when JN.1 dominated incidence (Fig. S1 of the Supplementary Appendix), and 12 February 2024. These databases encompass all laboratory and medically supervised SARS-CoV-2 testing, infection clinical outcomes, COVID-19 vaccination and demographic details within the country (Sections S1-S2).

Cases (SARS-CoV-2-positive tests) and controls (SARS-CoV-2-negative tests) were matched exactly one-to-two by factors that could influence the risk of infection, including sex, 10-year age group, nationality, number of coexisting conditions, number of vaccine doses, calendar week of the SARS-CoV-2 test, method of testing (polymerase chain reaction versus rapid antigen) and reason for testing (Section S3). Previous infection was defined as a SARS-CoV-2-positive test ≥90 days before the study test. Subgroup analyses estimating effectiveness against specifically symptomatic reinfection, and by vaccination status, were conducted.

Figure S2 and Table S1, respectively, show the study population selection process and characteristics. The overall effectiveness of previous infection in preventing reinfection with JN.1, regardless of symptoms, was estimated at 1.8% (95% CI: −9.3 to 12.6%) (Fig. 1). This effectiveness demonstrated a rapid decline over time since the previous infection, decreasing from 82.4% (95% CI: 40.9 to 94.7%) within 3 to less than 6 months after the previous infection to 50.9% (95% CI: −11.8 to 78.7%) in the subsequent 3 months, and further dropping to 18.3% (95% CI: −34.6 to 56.3) in the subsequent 3 months. Ultimately, it reached a negligible level after one year. The effectiveness was estimated at 49.1% (95% CI: 20.4 to 67.5%) during the first year and at −2.5% (95% CI: −13.5 to 9.0%) thereafter.

**Figure 1:**
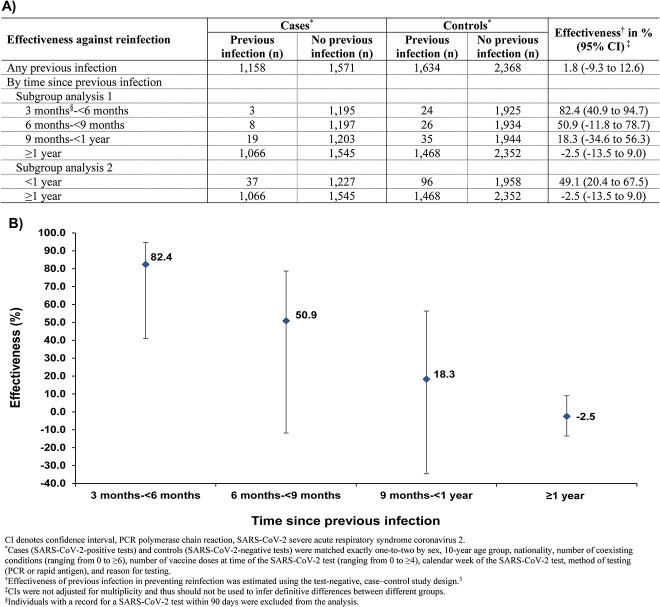
Protection against reinfection with JN.1, irrespective of symptoms, overall (A) and by time since previous infection (A and B)

The effectiveness against symptomatic reinfection with JN.1 demonstrated a similar pattern to that observed for any reinfection (Table S2). The overall effectiveness against symptomatic reinfection was −2.3% (95% CI: −14.4 to 10.3%). Subgroup analyses for unvaccinated and vaccinated individuals yielded results similar to those of the main analysis (Table S2). Limitations are discussed in Section S3. The study was reported according to the Strengthening the Reporting of Observational Studies in Epidemiology (STROBE) guidelines (Table S3).

The protection of natural infection against reinfection was strong among those who were infected within the last 6 months, with variants such as XBB*. However, this protection waned rapidly and was entirely lost one year after the previous infection. These findings support a considerable immune evasion by JN.1, and that this immune evasion led to the observed rapid waning of the protection against JN.1 (Fig. 1), a pattern for the effect of immune evasion first characterized for SARS-CoV-2 following the omicron variant emergence at the end of 2021.^4^^,^^5^

## Supplementary Material

Supplementary_Appendix_taae053

## Data Availability

The dataset of this study is a property of the Qatar Ministry of Public Health that was provided to the researchers through a restricted-access agreement that prevents sharing the dataset with a third party or publicly. The data are available under restricted access for preservation of confidentiality of patient data. Access can be obtained through a direct application for data access to Her Excellency the Minister of Public Health (https://www.moph.gov.qa/english/OurServices/eservices/Pages/Governmental-HealthCommunication-Center.aspx). The raw data are protected and are not available due to data privacy laws. Aggregate data are available within the paper and its supplementary information.
